# Fremanezumab and Non-High-Dose Galcanezumab for Comorbid Cluster Headache in Patients with Migraine: Three Cases

**DOI:** 10.3390/neurolint15010020

**Published:** 2023-02-24

**Authors:** Kenta Kashiwagi, Masahito Katsuki, Shin Kawamura, Senju Tachikawa, Atsuko Ono, Akihito Koh

**Affiliations:** 1 Department of Neurology, Itoigawa General Hospital, 457-1, Takehana, Itoigawa 945-0006, Japan; 2 Department of Neurosurgery, Itoigawa General Hospital, 457-1, Takehana, Itoigawa 945-0006, Japan; 3 Department of Neurosurgery, Hamamatsu University School of Medicine, 1-20-1, Handayama, Hamamatsu 431-3192, Japan

**Keywords:** anti-calcitonin gene-related peptide monoclonal antibodies, cluster headache, migraine, real-world, galcanezumab, fremanezumab, comorbidity

## Abstract

A new treatment option for cluster headache (CH) prevention is needed. Monoclonal antibodies (mABs) against calcitonin gene-related peptide (CGRP) ligands are used as a preventative treatment for migraine. Considering the CGRP’s role in the CH attack’s ignition and upkeep, fremanezumab and galcanezumab have been evaluated for CH preventative treatment. However, only high-dose (300 mg) galcanezumab has been approved for episodic CH prevention. We herein report three cases of migraine and comorbid CH with previous failures of preventive treatments. Two cases were treated with fremanezumab and one with non-high-dose galcanezumab. All three cases showed good results, not only for migraine, but also for CH attacks. This report suggests the efficacy of CGRP-mABs for CH prevention. Our cases differed from cases in the phase 3 trials of CGRP-mABs for CH prevention in two ways: first, our patients had both migraine and comorbid CH, and second, we used a combination of CGRP-mABs with preventative drugs, such as verapamil and/or prednisolone, to treat CH. Future accumulation of real-world data may prove the efficacy of CGRP-mABs for CH prevention.

## 1. Introduction

Cluster headache (CH) is one of the primary headache disorders. CH attacks are characterized by excruciating unilateral headache or facial pain accompanied by ipsilateral autonomic symptoms and restlessness or agitation [1]. CH patients suffer from a 60-month diagnostic delay despite the pain’s severity. The headache lasts for 15 to 180 min, often occurring in a circadian and circannual pattern [2,3,4]. Population-based studies calculated a lifetime prevalence ranging from 56 to 381 per 100,000 [5]. CH interferes with quality of life due to its severe pain attack, and its economic impact is significant [6]. Alcohol is a well-known trigger for CH [7]. A first-choice preventative drug for CH during cluster periods is verapamil [8] in Japan, according to the Clinical Practice Guideline for Headache Disorders 2021. Naratriptan [9,10] and ramelteon [11] are also considered as alternative prophylactic options [9,10]. Preventative treatments are used on a scheduled basis to reduce the frequency and intensity of headaches [12]. If it is ineffective, contraindicated, or discontinued due to side effects, the choice of preventative treatment can be difficult [13].

Monoclonal antibodies (mABs) against calcitonin gene-related peptide (CGRP) ligands are used as a preventative treatment for migraine [14]. CGRP is a 37-amino-acid signaling neuropeptide and is involved in migraine and other trigeminal nerve-related or facial pain disorders. CGRP-mABs are now widely used as epidemiological studies are conducted [15], and patient interest in treatment increases [16]. Considering that CH can be complicated by migraine headaches [17], that CGRP may relate to both CH and migraine [18,19,20], that trigger factors, such as alcohol, and treatment response to triptans are clinically similar [21], and that migraine headaches are accompanied by autonomic symptoms [4], CH and migraine headaches may overlap in their mechanism.

The pathophysiology of CH is based on the disruption of trigeminovascular connections, which results in the production of certain neuropeptides, such as CGRP [22]. Considering CGRP’s role in the CH attack’s ignition and upkeep [23], fremanezumab and galcanezumab have been evaluated for CH preventative treatment. High-dose (300 mg) galcanezumab has been proven effective for episodic CH prevention, but fremanezumab and non-high-dose galcanezumab have not been proven effective for CH prevention [24]. The efficacy of CGRP-mABs in CH without migraine was limited. However, no report has yet evaluated the efficacy of CGRP-mABs in the setting of coexisting CH and migraine, rather than pure CH.

Therefore, we herein report three cases of migraines without aura and with comorbid CH successfully treated using fremanezumab or non-high-dose galcanezumab for both migraine attack and CH attack prevention. We aimed to treat the migraine and consequently treated the CH as well. Our report suggests that fremanezumab and non-high-dose galcanezumab may be preventative treatments in patients with migraine and comorbid CH. Our three cases suggest that the pathophysiology may be different from past trials, which evaluated the efficacy of CGRP-mABs in pure CH. This article was previously posted to the ResearchGate preprint server in December 2022. All patients provided written informed consent.

## 2. Case Presentation

**Figure 1:**
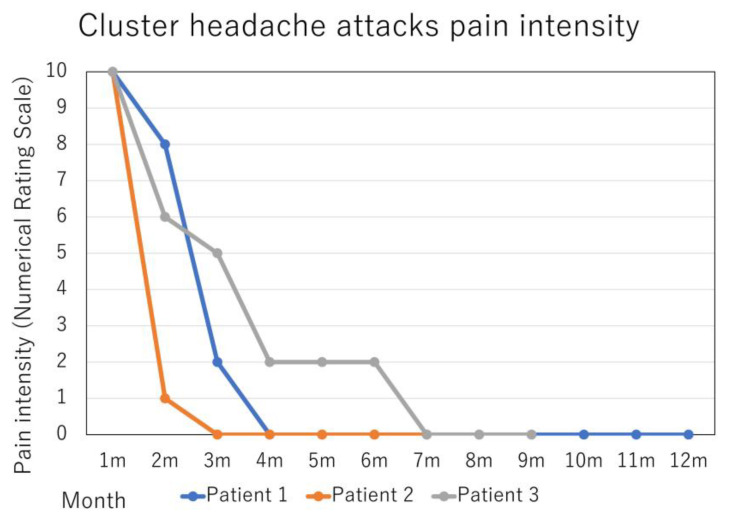
Change in the frequency of cluster headache (CH) attacks in the course of treatment.

**Figure 2:**
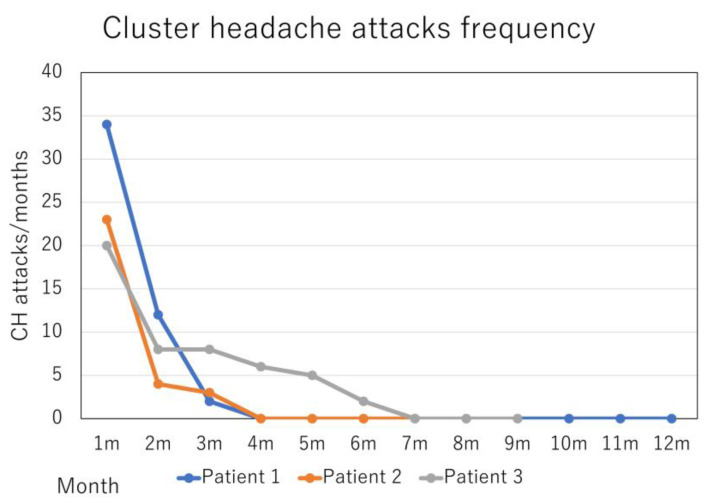
Change in the intensity of cluster headache (CH) attacks in the course of treatment.

The patients’ therapeutic results are summarized in Figure 1 and Figure 2.

Patient 1: A 35-year-old man presented with a 15-year history of excruciating, stabbing headache episodes with right orbital-periorbital localization, associated with ipsilateral autonomic symptoms such as ptosis, rhinorrhea, eyelid edema and agitation. The attacks ranged from 2 to 5 per day, and lasted 40–60 min, treated with a sumatriptan injection of 3 mg/0.5 mL. These attacks did not express in the period of remission, and the approximate 3-week cluster periods occurred 2–3 times per year. Verapamil 120 mg was not effective for CH prevention. He also reported migraine episodes five times per month, which resolved after over-the-counter combination analgesics use. Migraine was a comorbidity, not a co-occurrence with CH. The headaches were of moderate intensity, aggravated by routine physical activity, associated with photophobia, phonophobia, and nausea, and lasted 4–8 h. The headaches were located at the center of his forehead. Previously, he had started to take amitriptyline 20 mg as a prophylactic treatment, but stopped due to drowsiness. Based on the diagnosis of episodic CH (International Classification of Headache Disorders 3rd edition (ICHD-3) code 3.1.1) and migraine without aura (code 1.1), fremanezumab 675 mg was administered quarterly to prevent migraine attacks. After 3 months, monthly headache days (MHD) and monthly acute medication intake days (AMD) improved from 5 to 3 and 5 to 1, respectively. The frequency and pain intensity of CH attacks also improved from 34 to 2 and from 10 to 2 on the numerical rating scale (NRS), respectively. The patient reported sustained response for 12 months (five administrations of 675 mg fremanezumab) at the time of the follow-up appointment, and continued fremanezumab use. There have been no side effects of fremanezumab.

Patient 2: A 37-year-old man presented with a 12-year history of excruciating, stabbing headache episodes with left orbital-periorbital localization, associated with ipsilateral autonomic symptoms such as rhinorrhea, forehead sweating and agitation. He had had a suicide attempt. The attacks ranged from 2 to 4 per day and lasted 120–180 min, treated with a sumatriptan 50 mg intake. These attacks did not express in the period of remission, and the approximate 2-week cluster periods occurred 1–2 times per year. Verapamil 240 mg and prednisolone 40 mg were not effective as CH prevention. He also reported migraine episodes five times per month, resolved after sumatriptan 50 mg intake or over-the-counter combination analgesics use. Migraine was a comorbidity, not a co-occurrence with CH. The headaches occurred with bilateral temporal localization and moderate-severe intensity, were aggravated by routine physical activity, and associated with nausea, lasting 4 h. Previously, he had started to take valproic acid 200 mg as a prophylactic treatment, but stopped due to drowsiness. Based on the diagnosis of episodic CH (code 3.1.1) and migraine without aura (code 1.1), fremanezumab 675 mg was administered quarterly to prevent migraine attacks. After 3 months, MHD and monthly AMD improved from 5 to 0 and 5 to 0, respectively. The frequency and pain intensity of CH attacks also improved from 23 to 3 and from 10 to 2 NRS, respectively. The patient reported sustained response for 7 months (three administrations of 675 mg fremanezumab) at the time of follow-up appointment, and continued fremanezumab use. There have been no side effects of fremanezumab.

Patient 3: A 59-year-old man presented with a 12-year history of severe, stabbing headache episodes that were localized to the left orbit and the periorbit, and were accompanied by ipsilateral autonomic symptoms such as rhinorrhea, forehead perspiration, eyelid edema, and psychomotor agitation. He had made a suicide attempt. Attacks ranged from 2 to 4 per day and lasted 180 min. Sumatriptan 50 mg or a 3 mg/0.5 mL injection was used to treat them. They did not, however, exhibit strong therapeutic responses. For the previous 5 years, there had been no remissions. Prednisolone 40 mg and verapamil 240 mg had no impact on CH prophylaxis. He also stated that he experienced migraines four times each month. Migraine was a comorbidity, not a co-occurrence with CH. He took sumatriptan 50 mg or a combination of over-the-counter painkillers. The headaches were severe, localized to the left temporal region, aggravated by normal physical activity, and lasted for 8 h. They were also accompanied by photophobia, phonophobia, and nausea. He had previously taken 400 mg of valproic acid as a preventative measure, but he discontinued it due to skin rash and sleepiness. Galcanezumab was used to stop migraine attacks based on the diagnoses of migraine without aura (code 1.1) and chronic CH (code 3.1.2). Galcanezumab 120 mg was administered subcutaneously once per month after a 240 mg loading dose. MHD and AMD both improved from 4 to 2 and 10 to 2, respectively, after 3 months. The frequency of CH episodes decreased from 20 to 8, and the pain level decreased from 10 to 5. At 7 months, chronic CH had entered remission. The patient reported ongoing use of galcanezumab, a sustained response that had lasted for 10 months at the time of the follow-up session. There have been no side effects of galcanezumab.

## 3. Discussion

We report three cases of migraines without aura and comorbid CH successfully treated using fremanezumab or non-high-dose galcanezumab for CH attack prevention. We aimed to treat the migraine and consequently treated the CH as well, leading to the reduction of frequency and intensity of CH. We herein discuss why CGRP-mABs might be effective for CH and describe the previous trials regarding using CGRP-mABs for CH prevention.

Differences and similarities have been reported between CH and migraine [21]. As to the differences, migraine mostly affects women (women to men ratio: 3:1), whereas CH is more common in men (men to women ratio: 4.3:1) [7]. Individuals with CH have a prevalence of migraines similar to that of the general population [25]. Furthermore, whereas migraine headache localization might shift or be bilateral, CH attacks are frequently side-locked and usually occur on one side. A CH attack lasts between 15 and 180 min, and there may be several attacks per day. In contrast, a migraine attack lasts between 4 and 72 h, and recurrence is defined as a headache that appears within 22 h of the migraine’s initial successful treatment [26]. Regarding the similarities, both headache disorders have autonomic symptoms [4], and CGRP may be related to their pathophysiology. Furthermore, on rare occasions, patients may describe an intermediate phenotype that combines characteristics of both primary headaches or has concomitant CH and migraine [27]. Whether these two diseases should be treated as a spectrum is under debate.

The two keys of CH pathophysiology are the hypothalamus as the central structure and generator of CH and the trigeminal nerve as the peripheral structure for pain and autonomic symptoms. CH’s circadian and circannual clustering of attacks may suggest a relationship with the endogenous biological clock. The hypothalamus is responsible for circadian rhythm, and neuroimaging studies have shown functional and structural alternations of the hypothalamus in CH patients [28]. As for the mechanism by which trigeminal overexcitation causes pain and parasympathetic activation, it is thought that the trigeminal nerve activity is heightened, and that this excitement extends to the superior salivary nucleus, resulting in excitation of parasympathetic nerves from the pterygopalatine ganglion to the large intracranial vessels, lacrimal gland and nasal mucosa. A series of autonomic symptoms, such as running tears and nasal discharge, are produced. Furthermore, the increased release of CGRP and other substances upon stimulation of the trigeminal ganglion and the increase in CGRP in jugular venous blood during CH attacks suggest a close involvement of the trigeminal nerve and CGRP in CH [24]. The hypothalamus, trigeminal nerve and CGRP are closely involved in both migraine [29] and CH. Migraine and CH may also be accompanied by clinically overlapping findings; up to 46% of CH patients can show migraine-like features, and migraine patients can have typical trigeminal autonomic symptoms [17]. Additionally, 15.6% of CH patients have comorbid migraine, although comorbid CH in migraine cohorts has yet to be investigated [30]. Furthermore, 42.4% of migraine headaches are accompanied by autonomic symptoms [4]. There may be some commonality in pathophysiological pathways, but there are also significant variances, according to demographic, genetic and temporal trends. Common pharmacological triggers point to a shared anatomical and pathophysiological substrate, but as CH attacks are initiated more quickly than migraine attacks, the signaling cascades involved in attack initiation may vary [21]. Taken together, however, CGRP functional blockade may alleviate neurogenic inflammation and reduce pain pathway sensitization in both migraine and CH; considering this, CGRP may play an important role in CH.

Fremanezumab is effective in preventing episodic and chronic migraine. In the episodic CH prevention study (NCT02945046), fremanezumab dosages of 900/225/225 mg for the high-dose arm, 675 mg/placebo/placebo for the low-dose arm, and a placebo arm were compared. The dosing schedule was 0/4/8 weeks. In 169 cases, there were no differences in the weekly average number of CH attacks between the arms during the 4-week period (fremanezumab high dose: 7.6 attacks/week vs. fremanezumab low dose: 5.8 attacks/week vs. placebo: 5.7 attacks/week).

Galcanezumab is used for the prevention of episodic and chronic migraine. One phase 3 randomized placebo-controlled trial (NCT02397473, CGAL study) investigated the efficacy of galcanezumab (subcutaneous injection of 300 mg/month for 2 months) for episodic CH prevention [31]. Of the 106 cases, galcanezumab effectively reduced CH attack frequency across week 1 to week 3 (galcanezumab: 8.7 attacks/week vs. placebo: 5.2 attacks/week, *p* = 0.036). There are no studies regarding the use of non-300-mg galcanezumab for CH prevention, but there is a meta-analysis of randomized controlled trials regarding different doses of galcanezumab versus a placebo in patients with migraine and CH [32]. Based on these studies, galcanezumab is available as a preventative treatment for CH in the United States and approved by the Food Drug Administration [33,34]. On the other hand, the European Medicines Agency rejected the approval of galcanezumab (100 mg × 3/monthly subcutaneously) for the prevention of episodic and chronic CH. This is because results from the single study of patients with episodic CH did not clearly show that galcanezumab is effective for preventing CH attacks. Therefore, the benefits of galcanezumab in preventing attacks in episodic CH patients did not outweigh its risks. In these countries, verapamil at high dosages is still used, but its use is limited by its cardiovascular risks, such as atrioventricular block [1].

Although not a randomized control trial, a real-world experience with 240 mg of galcanezumab for the preventive treatment of CH has been reported [35]. Galcanezumab was begun a median 18 days (range 1–62 days) after the current bout began in 47 patients with episodic CH, and 4 patients (10.8%) received a second dose of the medication. In 33 patients who kept a headache diary, 78.8% had weekly CH attacks that were 50% or less frequent than they had been at the beginning of the study. It was concluded that galcanezumab 240 mg dose, with or without conventional therapy, was regarded as efficacious and safe in clinical settings for the prevention of CH.

As additional real-world evidence, retrospective data from 22 patients with chronic CH who had at least one dosage of CGRP-mABs and kept track of their attack frequency in a headache diary were gathered from eight headache clinics [36]. Significant reductions in acute headache medication use on a weekly basis and pain intensity during attacks in the first month of the study were observed. Three erenumab 70 mg, erenumab 140 mg and galcanezumab 240 mg were included. This study suggests that erenumab, an anti-CGRP-receptor antibody, has the potential to be a preventative drug for CH. The efficacy of erenumab in chronic CH has been tested (NCT04970355).

Study design for episodic CH preventative therapy is difficult regarding the following points. Spontaneous remission of CH can occur. Irregularity and changes in the onset and duration of cluster periods also exist. Therefore, negative study results regarding CGRP-mABs for episodic CH are not absolute. They could be meaningful if the study design and cases were rigorously examined. Additionally, our cases were different from the cases in the phase 3 trials in two ways: first, our patients had both migraine and comorbid CH, and second, we used a combination of CGRP-mABs and preventative drugs, such as verapamil and/or prednisolone, for CH. Furthermore, it is important to balance the shift away from randomized control trials, which provide a mathematical understanding of how medications are applied to a particular illness, with the adaptability of clinical practice, as seen using real-world evidence data [37]. Therefore, it is hoped that the future accumulation of real-world data may prove the efficacy of CGRP-mABs for CH, for which treatment options are scarce.

## 4. Conclusions

We report three cases of migraines without aura and with comorbid CH successfully treated using fremanezumab or non-high-dose galcanezumab for CH attack prevention. We aimed to treat the migraine and consequently treated the CH as well. The frequency and intensity of CH were improved by CGRP-mABs use.

## Data Availability

Not applicable.
